# Supplementary material for: MetaRibo-Seq measures translation in microbiomes
Source: Nat Commun. 2020 Jun 29;11:3268. doi: 10.1038/s41467-020-17081-z (PMC7324362; doi:10.1038/s41467-020-17081-z)
Supplement: Supplementary file 10 — Supplementary Data 7 [file 41467_2020_17081_MOESM10_ESM.zip › File2/Confidence_High_Taxonomy/335282_out.krona.html]

Javascript must be enabled to view this page.

members
magnitude
magnitudeUnassigned
count
unassigned
taxon
rank

335282\_out

2

2
2
superkingdom

phylum
2
1224

class
28216
2

order
2
80840

995019
2
family

genus
40544
2

species
40545
2

SRS015264\_contig\_number\_23009SRS077086\_contig\_number\_12424
